# Medical Education

**Published:** 1887-02

**Authors:** P. P. Wells

**Affiliations:** Brooklyn, N. Y.


### HOMŒOPATHIC PHYSICIAN,

A MONTHLY JOURNAL OF MEDICAL SCIENCE.

"If our school ever gives up the strict inductive method of Hahnemann, we are lost, and deserve only to be mentioned as a caricature in the history of medicine."—CONSTANTINE HERING.

Vol. VII. FEBRUARY, 1887. No. 2.

### MEDICAL EDUCATION—WHAT IS IT, AND WHERE AND HOW IS IT TO BE OBTAINED?

P. P. WELLS, M. D., BROOKLYN, N. Y.

No subject is oftener or more earnestly discussed when doctors are assembled than is this, and always in the interest of a felt want of something better than the present experience is furnishing, or than the past has given us. They insist, and with apparent sincerity, on the duty and necessity of this better, and no one of them more earnestly than those who stand in the place of public teachers of medical sciences. The members of our college faculties, they seem to feel more and *talk* more of this need than others, but never tell us how or where this felt want is to be supplied. It is worthy of remark that the improvement advocated is, as presented by these professors, to be a something wrought by pupils themselves. They never seem to have had an inkling of thought that this better is to come, if it comes at all, from *better teaching.* Present teaching or teachers they are not supposed to charge with deficiency, but the pupil is to be spurred to greater diligence, and taxed for more time given to present teaching and teachers; and yet, after all this very proper talk of this real want, their pupils are sent out just the same living witnesses to this great need as before. They have not found the needed better. A kind of consciousness of this fact seems, at times, to peer out in the troubled, restless minds of these teachers, which seeks relief in change, as though any change must be for the better. And changes are made, and some of them, if not so plain in their disclosure of utter ignorance of the true nature of this great want, would be not a little funny, by reason of the *bizarre* character of the resorts which constitute the changes in which these simple ones seem to have prided themselves, and from which they seem to have hoped that good would come to their classes somehow and in some way not very clearly discerned. An example of this appeared a little more than a year ago, when it was announced by one of our colleges (we are concerned now only with *homœopathic* medical colleges) that it had made it the duty of one of their professors to teach the manner of preparation and *use* of the three pet p's of old physic, pukes, purges, and poultices. Surely it could have been no less than heathen blindness and ignorance which could have supposed, for a moment, that these abominations of old physic could add aught of value to a homœopathic curriculum, or could, by any possibility, become a part of one.

A part consciousness of this, and of the other fact that theirs was supposed to be a *homœopathic* college, seems to have possessed this faculty, for at the same time they announced this supposed improvement of their curriculum, this which added the three now notorious p's, they also promised an equal amount of time and thought would be given to teaching Hahnemann's *Organon*. This codified God's law of healing seems to have had an importance in their minds, as an element of medical teaching and education, just equal to that of these three p's, for equal time was to be given to each. Each was to have *one* lecture a week through the session. We do not know how it fared with the p's, but we are informed by a pupil of the class to whom this promise was made that there was just *three* attempts made to talk of the *Organon*, that these were short, and that these talks, and notably the last one, were miserable failures. How could it have been otherwise with any member of a faculty who could conceive of this grossest of all absurdities, this of introducing these p's into a *homœopathic* curriculum as an added improvement? The conviction is irresistible that such a faculty must have been wholly blind as to the nature of the elements of the law they had been set to teach, and this conviction is confirmed by their failure in efforts to teach the *Organon*. This outcome is a response to our inquiry, when the American Institute, in June last, called on our colleges to teach the *Organon*, we, in view of the personnel of our college faculties, asked, "Where is the *vind to come from?*"* We

* Echo, in this failure, answers—from *nowhere!*

thought there was no more breath in them for this duty than there was in asthmatic Old Weller, wherewith to execute the called-for blowing. This faculty, certainly, when attempting the promised teaching, were apparently struck with a paroxysm of scientific asthma, and there was no breath in them for this effort, and we were not surprised. As they didn't *know*, of course they could not *teach*.

That this particular faculty does not know is further shown by the following utterance of one of its members in its present session:

> "Gentlemen, don't fall into that silly, ridiculous, narrow-minded idea of thinking that after having given your remedy, you can sit down and fold your hands, and say that everything has been done for your patient. Everything has not been done. Don't stop until you have exhausted every means known to you. Homœopathy is a grand, good thing, gentlemen, but there are lots of good things outside of Homœopathy. You may be called to a child in convulsions. In this any old nurse could give you points, if you did not already know them."*

Before a proper notice of this paragraph can be taken, there are some things to be borne in mind. First: This was the utterance of a teacher of materia medica in a *homœopathic* medical college. Second: It was given to a class who had come to this College to be taught the science of *homœopathic* practical medicine. Third: The College is the same where, after three attempts to teach this science as given us in the *Organon*, it was abandoned. Couldn't do it, was the practical outcome of this abortive endeavor. Fourth: That the utterance is from the professor of the *three p's*. Fifth: When this professor says, "*After giving your remedy*," he means after giving the *homœopathic* remedy for the case. He, being a professor in a *homœopathic* medical college, and ostensibly engaged in teaching the medicine which the title of his College proclaims, could have meant no other. Sixth: The homœopathic remedy is the *specific* for the cure of the case. Seventh: The *specific* curative is equal to *all* the demands for the relief of any curable case. Eighth: The specific needs no aids in the performance of its office from "*old nurses*" or any other source. Ninth: The members of this class and others, including this professor of p's, may learn, if they have

* Why not teach these to the class, then and there, as was his duty, instead of leaving its members to find them out from "old nurses." Are members of this faculty inferior to these respectable old people in knowledge of proper clinical resorts? Is this a proclaimed inferiority of this faculty to that class of respectable and useful old people? With the two, as to a knowledge of homœopathic philosophy and practice, we should judge, from this sentence, that a comparison of the two would disclose very little difference.

the grace to try it and the knowledge necessary for the trial, that after having given the specific remedy, his highest *wisdom* will be employed in letting it alone. He may as well "sit down and fold his hands" as anything else. He has done his *whole* duty when he has found his specific remedy and given it, and it is his imperative duty to find this and give it. Having done this and *waited* the result, he may find this waiting neither so "*silly*" nor "*ridiculous*" as, in his ignorance, he may have supposed.

But it is possible one of these pupils may inquire, How am I to know I have found the specific for my case? The question is a very proper one. We may refer the inquirer to his professor for the proper answer. To answer this question, or rather to teach this inquirer to answer it for himself, is just that for which he has been put into the official chair he occupies, and it is but a scurvy dodge when, to avoid teaching the needful answer to this question, and of that which will enable him to find his specific, he refers his class to some "*old nurse.*" If this be the outcome of his teaching of specific medicine, we see no great loss that can come to any one, or to any interest, if he should turn his class over to "old nurses" for whatever else they may need to know, and he "should step down and out." In view of this utterance of his, we cannot but regard the one who waits, not as the most silly and ridiculous one on the scene, for in these he must yield long preference to this would-be teacher of a science of which, in this one sentence, he gives so great and clear evidence of utter ignorance.

Having disposed of the "*silly*" and "*ridiculous*," how is it of the "*narrow-minded*"? To whom does this most damaging term attach with greatest force and tenacity, when the question is the clinical administration of that "*grand, good thing*" this professor declares Homœopathy to be? What is this grand and good thing? It is just this and nothing less, a law enacted by divine power when it created men subject to sickness, for his deliverance from its pains and dangers to life. It is this law and its corollaries, so given of God, and a *science* of therapeutics based on these, developed by the insight, wisdom, and industry of man. This law and this science have a breadth of existence and applicability co-extensive with the whole domain of human diseases. A law of relationship of sicknesses and their curatives, so originating, could have no less extent than this. Omniscience and omnipotence have so created and determined this relationship, and the mind which comprehends and embraces this relationship is not narrow, and cannot be rightly charged with narrowness, when found loyally obeying its precepts. On the contrary, that mind is "*narrow*" which, before this law and this science, is found to be incapable of grasping their scope or dealing with the principles loyalty to these involves. That mind is "*narrow*" which, in practical duties, is compelled, by inability to grasp these principles, to abandon them and go after outside resorts, of whatever kind, with which they may be more familiar, and these may, at times, be found to be "*old nurses.*" This "*narrow-minded*" imbecility it is which leads to the slip-slop practice which characterizes those who claim the freedom to give their patients whatever "*they think will do them good!*" thus exalting their weakness and blindness above God's law! This is the narrow mind which, failing to comprehend the nature and extent of this law, and therefore failing of the successes which a legal compliance with its demands assures, runs off after those "other" supposed "good things outside of Homœopathy," which, therefore, have no place in the science of therapeutics, and no relation of curative to the case under treatment. These are the narrow-minded ones who, by this disloyal course, are not only made narrower by it, but weaker before all clinical problems they may be called to solve.

Then, as to an answer to the first question in our title of this paper—What *is* Medical Education? As, when speaking of colleges, we had reference only to *homœopathic* medical colleges, so here, when questioning as to what constitutes a medical education, it is a *homœopathic* education of which we mean to speak. This can only consist of a knowledge of homœopathic law and its corollaries, together with a knowledge of how to apply the science of therapeutics, founded on these, for the cure of the sick. Any education which leaves out a knowledge of these is not a medical education in the true sense of these words. This education must contain a knowledge of the true philosophy of sickness, and also of the nature of the curing agents, and of the rules for the use of these for the solution of clinical problems. A medical education in this sense is an education which enables one, before a case of sickness, to put his hand on the specific agent which will cure it. To be able to do this according to law is the objective, and only objective, of this education.

And then the second question—Where can this education be obtained? And in response we are both sorry and ashamed to say there comes to us little else than echo, and this only answers —*Where?* Why is it that this is so? Are there not incorporated institutions, and more than enough of them, which have been created for this one purpose, to give this education to those who seek it? Then why, when we ask where can this be obtained, are we not told that in this and that place, where these institutions are located, this education can be had? Is there any other *raison d'être* for these institutions but that in them that which constitutes this education might be taught there? Then why is it not? Why, when called for a knowledge of the place, is it left to solitary old echo alone to answer? Why are institutions incorporated for the teaching of Hahnemann's Homœopathy given over to teaching how to prepare and use pukes, purges, and poultices, and other knowledges more in repute and in current use by old physic, to the exclusion of a knowledge of the philosophy of specific prescribing? Why should it have been that when a member of one of these faculties, more intelligent in the philosophy of specific medicine than his fellows, and seeing clearly the indispensable necessity of this to the class of pupils there gathered, when offering to teach them this, should he have been told by a fellow-member, "*If you attempt this the faculty will take your head off*"? Why, but that his knowledge of this philosophy which enabled him to perform what he had offered to do would, if brought into action, be a constant rebuke to his associates who were so conspicuously destitute of it. Why this threat but that they saw the need of the exclusion of this philosophy as a defense of their own ignorance of it. It could, by no possibility, be an injury to the pupils. Indeed, want of a knowledge of this was just what had gathered them in this College, that there this want might be supplied. It could not hurt them. It could not hurt the public that these young men should come out from this College to serve them in their sicknesses armed with a knowledge of how to find the specific cure for each case of their sickness. This knowledge it was that this College was created to impart to pupils, for their benefit, and that of this creating and trusting public. It could only hurt this faculty, and this only by the contrast of the work of this one homœopathic member with that of his fellows, into the work of which Homœopathy has never been wrought, and in which it is never found. This was so apparent to the member to whom the offer to teach this philosophy was made, and such was his knowledge of the character and status of his associates, that he assured this one intelligent member that if he attempted this their outcry would be at once, "*off with his head.*"

Why is it that in all our so-called *homœopathic* colleges there is no attempt to teach this philosophy, with two solitary exceptions, and in these exceptions by only one member of the faculty of each? And why is it that in one of these the member so teaching is made to feel that he labors in a fighting opposition to his work, and that the other should be exposed by his so doing to the danger of *losing his head?* Is it so that teaching that philosophy, for the promulgation of which these colleges were created, has become a crime? Against what is this a crime, except against the selfishness of ignorance?

Before the fact of this neglect on the part of these faculties to teach the philosophy and practice of the Homœopathy of Hahnemann, and their almost unanimous opposition to this being taught, are we wrong when we charge them with *false pretense* and *malfeasence* in their official work, when this is so largely an imitation of the teachings and work of old physic? These are crimes in dealings and interests extra professional. Are they less so when colleges called homœopathic attract pupils to their lecture rooms by their titles, and then give them poor imitations of old physic, with a mixture of hate for and opposition to that for which these institutions had been created. Instead, we find there the current effort is not to teach but to destroy these and all devoted to them.

We think we may be pardoned if, in now dismissing this subject, we call the attention of these faculties to three other p's than those the silly introduction of which into a homœopathic college curriculum has made one of their number so conspicuously ridiculous. These other three, we beg to assure these faculties, though by them so shamefully abused in their past history, have rights which are every way worthy of their respect; nay, we may go further, and express the hope that if present abuse is continued these faculties may be *compelled* to respect them. We refer to the *pupils* assembled in their lecture rooms that they may be taught; to *patients* who may apply to these, now pupils but hereafter doctors, to be cured of their sicknesses; to the *public* who have given to these colleges their corporate rights. The pupils have a right to demand that they be taught that knowledge of the philosophy and practice of specific medicine for which they came to these faculties, that they might by them be taught. They have a right to be taught that Homœopathy is not a question of *quantities* but of *philosophical principles*, and that they be taught these *principles*. The *patients* have a right to demand a knowledge of these by the doctors they call for their relief, who hold diplomas signed by these faculties, certifying that they have been so taught, when the fact is they have not. The *public* who have created these institutions for their own good, and have given to them corporate rights, have a right to demand that this lying on parchment shall stop. That these faculties either teach the philosophy and practice they were appointed to teach, or to "*step down and out*," and give place to other and better men, who can and will perform the duties owing to these other three p's, which present faculties have hitherto so shamefully neglected.

If we now repeat the second question in our title—Where is this education, which embraces a knowledge of the philosophy and practice of specific medicine, to be obtained? experience of the facts in the case replies to the voice of echo—*Almost* NOWHERE!

